# PhthisisBioMed Artificial Medical Intelligence: Software for Automated Analysis of Digital Chest X-ray/Fluorograms

**DOI:** 10.17691/stm2023.15.4.01

**Published:** 2023-07-28

**Authors:** Y.T. Gogoberidze, V.I. Klassen, M.Y. Natenzon, I.A. Prosvirkin, A.V. Vladzimirsky, D.E. Sharova, V.V. Zinchenko

**Affiliations:** Senior Development Engineer; PhthisisBioMed LLC, 135 Karla Marksa St., Chistopol, Republic of Tatarstan, 422980, Russia;; Professor, General Director; RK Vector JSC, 135 Karla Marksa St., Chistopol, Republic of Tatarstan, 422980, Russia; Board Chairman; PhthisisBioMed LLC, 135 Karla Marksa St., Chistopol, Republic of Tatarstan, 422980, Russia;; Senior Researcher; Russian Academy of Sciences, 14 Leninsky Prospekt, Moscow, 119071, Russia; Chairman of the Board of Directors; Scientific and Production Association National Telemedicine Agency, Office 8, 9 Demyana Bednogo St., Moscow, 123423, Russia;; IT Director; RK Vector JSC, 135 Karla Marksa St., Chistopol, Republic of Tatarstan, 422980, Russia;; Deputy Director for Scientific Research; Scientific and Practical Clinical Center for Diagnostics and Telemedicine Technologies of the Moscow Department of Health, Bldg. 1, 24 Petrovka St., Moscow, 127051, Russia; Head of Innovative Technologies Department; Scientific and Practical Clinical Center for Diagnostics and Telemedicine Technologies of the Moscow Department of Health, Bldg. 1, 24 Petrovka St., Moscow, 127051, Russia; Head of Clinical and Technical Trials Sector; Scientific and Practical Clinical Center for Diagnostics and Telemedicine Technologies of the Moscow Department of Health, Bldg. 1, 24 Petrovka St., Moscow, 127051, Russia

**Keywords:** artificial medical intelligence, PhthisisBioMed AI service, neural networks, thoracic organ diseases, CDSS, URIS UMIAS

## Abstract

The scope of diagnostic medical examinations increases from year to year causing a reasonable desire to develop and implement new technologies to diagnostics and medical data analysis. Artificial intelligence (AI) algorithms became one of the most promising solutions to this problem and proved themselves in the course of mass practical application. During the three-year Moscow experiment started in 2020, the possibility was achieved to develop methodologies of AI use and to successfully implement it into the regional level healthcare system.

In this article, the authors share their experience in developing a medical AI service using the example of PhthisisBioMed AI service and the results of its application in real clinical activities environment. This AI service has shown its quality and reliability confirmed by technological monitoring.

Clinical trials of PhthisisBioMed AI service were conducted on a specially prepared verified data set (n=1536) considering epidemiological indicators of the thoracic organs major diseases prevalence. The mean sensitivity of the service was 0.975 (95% CI: 0.966–0.984).

PhthisisBioMed medical AI service is registered as a medical device (medical device registration certificate No.RZN 2022/17406 dated May 31, 2022) and is actively used in the Russian Federation as a diagnostic tool to reduce the burden on radiologists and to accelerate the process of medical report obtaining.

## Introduction

With healthcare development and increasing medical services scope primarily caused by implementation of mass clinical examination and primary care, the number inevitably increases of diagnostic data to be considered to make a diagnosis and to prescribe the treatment.

According to the Rosstat data, the number of annual fluorographic examinations increased from 70 million people in 2017 to 87.7 million in 2023 (plan of the Russian Ministry of Health) [1], while the total number of radiologists (phthisiatricians) decreased by an average of about 100 people per year.

Processing vast amounts of information to help individual patients requires to switch to digital healthcare and to create clinical decision support systems (CDSS) using big data analytics to optimize care delivery.

Creation of medical artificial intelligence (MAI) products with deep computer-based training and high technical performance is only possible if medical professionals and IT specialists interact closely.

The widespread practical application of MAI has made it clear that today’s medical community is sharply polarized in its trust in artificial intelligence (AI): from sharp, total rejection to unconditional faith and lack of minimal skepticism. Attempts to automate, almost “mechanize” the physician’s work process often do not yield the desired result due to a misunderstanding of the range of tasks that MAI can solve and the limitations inherent in the very AI concept. The desire of some specialists to relieve themselves as much as possible of their routine workload often leads to a constant unconscious agreement with MAI conclusions.

This article discusses CDSS implementation and mass exploitation related to a specific intelligent medical service based on AI, and in light of the large ongoing Moscow experiment, which includes the mentioned service.

## PhthisisBioMed medical AI service

### Model and architecture

Medical AI service is a software product providing the user with a standalone digital medical service. It is a complex multi-unit structure containing neural network algorithms trained to perform a diagnostic task as a rule.

PhthisisBioMed AI service began to develop PhthisisBioMed LLC in 2014 and has gone through several long cycles of development, improvement, and operation. Currently, version 3.3 is in operation. The core of the system is its intelligence unit.

#### Intelligence unit

**Figure 1.**
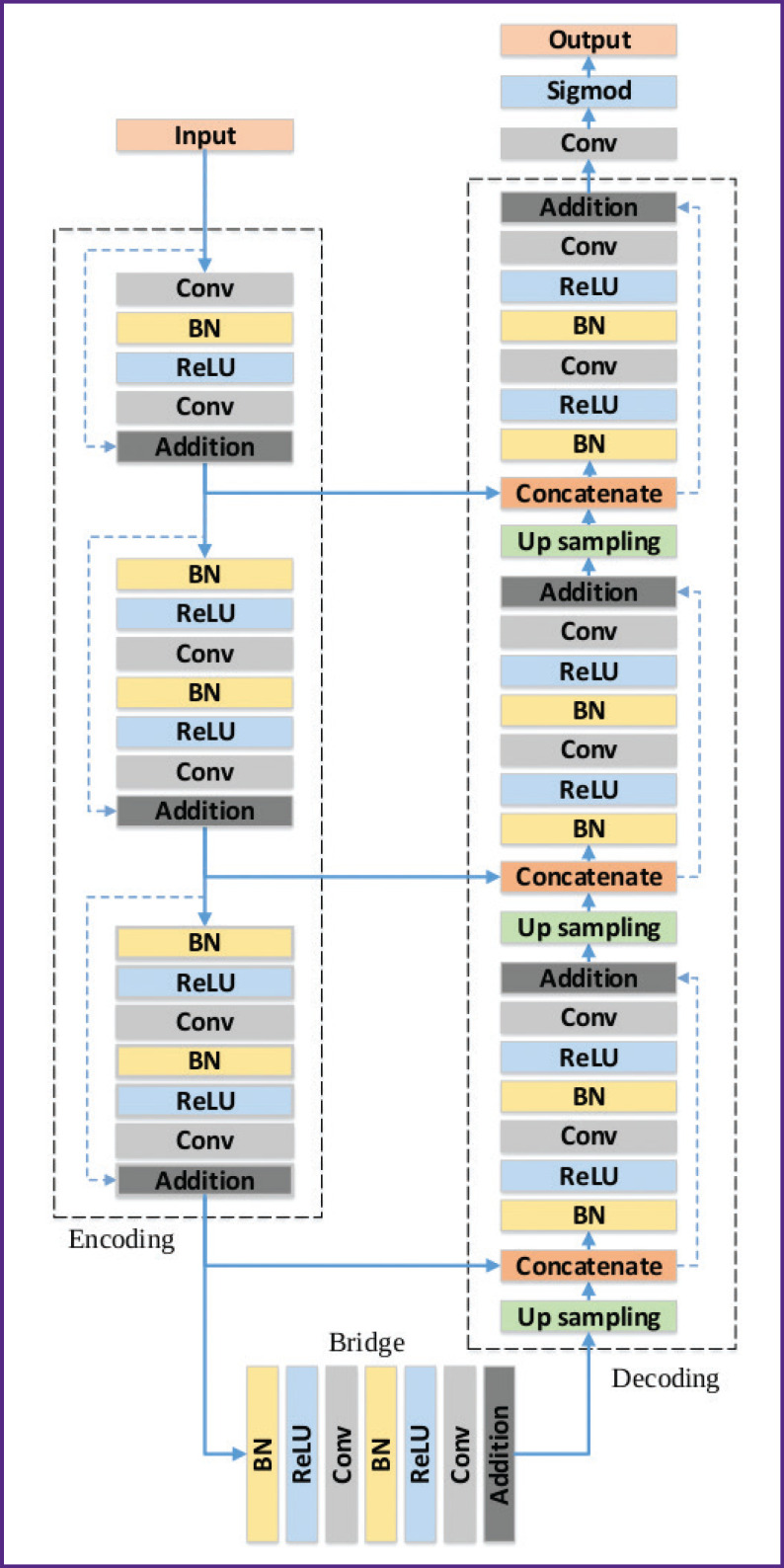
ResUNet architecture

PhthisisBioMed MAI intelligence unit is based on the model of a deep fully convolutional neural network adapted to detect and localize pulmonary pathologies. The basic architecture for PhthisisBioMed MAI neural network was the U-Net architecture, which was further modified to better fit the task. This issue is discussed in more detail in the authors’ previous publication [2]. We shall present here the general data of ResUNet model (Figure 1). The intelligence unit consists of three such neural networks, forming an ensemble.

**Figure 2.**
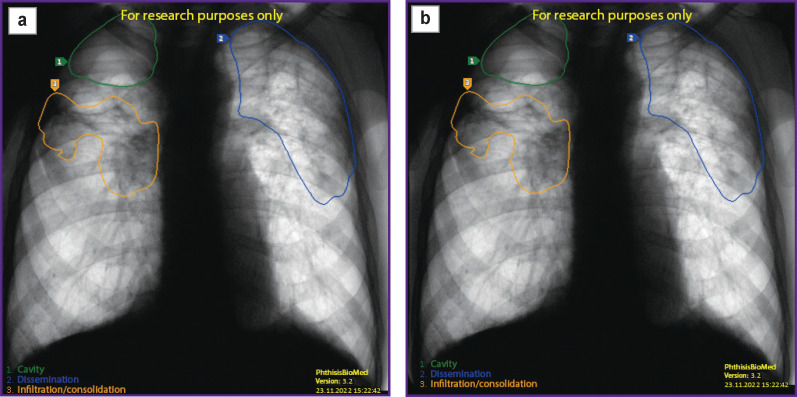
Result of PhthisisBioMed artificial medical intelligence use: (a) original image submitted for analysis; (b) analysis result

An additional element is the multilayer classifier, which is also a deep-training model. The classifier performs the function of separating and recognizing the types (classes) of pathologies detected and localized in the images which are analyzed by an ensemble of localizers. The basic model of the classifier is DenseNet201 [3]. Figure 2 shows the result of processing a fluorographic image using PhthisisBioMed AI service.

The next most important component of AI service structure is the superstructure unit.

#### Superstructure unit

One of the problems of medical image analysis is standardization lack for creation of the images themselves. Even if formal regulations exist, they are often frameworks.

Lung scans taken while the patient is standing, lying, or sitting have different specific properties, and the current clinical state is also important. It is also worth remembering that X-ray and fluoroscopy machines from different manufacturers have different properties and options. All this complicates image analysis using neural network models alone to search for abnormalities and requires provision of the service with additional algorithms which could account for these machine features.

The superstructure unit is used to preprocess the image before sending it for analysis. This involves the following manipulation of the image:

search for the region of interest in the image (in this case, the lungs), which implies intelligent (using a neural network) and algorithmic segmentation of the organ;

preliminary analysis of properties of the image itself, i.e., algorithmic determination whether the image is negative or positive along with neural network analysis: whether the image itself is a correct sample for analysis (whether the image is a radiographic image of the chest in frontal projection).

PhthisisBioMed service uses several mechanisms for data preprocessing: filtration, brightness characteristics of the image detection (negative/positive, range), area of interest segmentation.

**Figure 3.**
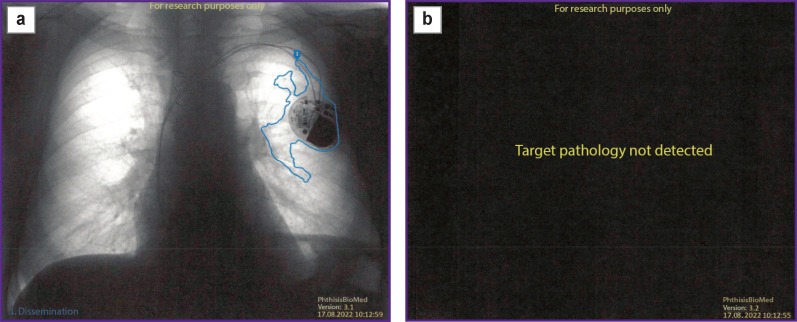
Use result of the superstructure model designed to minimize false activations on foreign bodies: (a) intelligence block activation without superstructure neural network recognizing activation on foreign bodies; (b) result after postprocessing

In addition, an important aspect is the detection of foreign bodies in chest images like pacemakers or surgical sutures along with any foreign objects caught in the image like necklaces. These objects often cause false positives in the intelligence unit neural networks. To minimize such triggers, a special superstructure model (Figure 3) is used to recognize and eliminate false activations generated by the intelligence unit neural network.

The last unit is for interaction with the client.

#### Client interaction unit

This unit is a set of measures, algorithms, and interaction protocols to automate the service operation during a real clinical process. In general, the unit allows to create uninterrupted lines of examinations processing performed in parallel and remotely in relation to the physical location of the service server itself. With this unit, it is possible to perform remote paralleled analysis and support physicians not only in individual medical institutions, but also in medical infrastructures on a regional scale in real time.

### Training

It is impossible to achieve AI high diagnostic accuracy without a training sample labelled with high confidence. According to a study by Seoul National University researchers [4] on the assessment of radiological experts diagnostic accuracy, the area under the ROC curve for qualified experts is 0.781–0.907 with a confidence probability of 0.95 for localization tasks.

**Figure 4.**
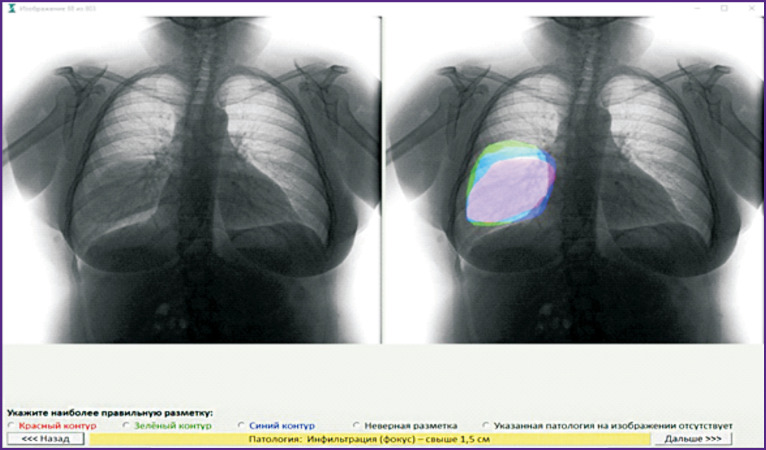
Software for monitoring the results of preliminary examination labelling

To assess the diagnostic accuracy of the expert physicians who labelled the sample for PhthisisBioMed service computer-based training, a preliminary study was conducted on overlapping samples. Three physicians labelled the same sample independently. Then, arbitrators from the Central Tuberculosis Research Institute (Russia) monitored the labelling results. For each pathology found, the arbiter used a special software (Figure 4) to decide whether the pathology was present at the image and, if so, who of the three labelling physicians located it most accurately.

Two district-level and one regional-level examining physician with more than 15 years of experience participated in the labelling.

**Figure 5.**
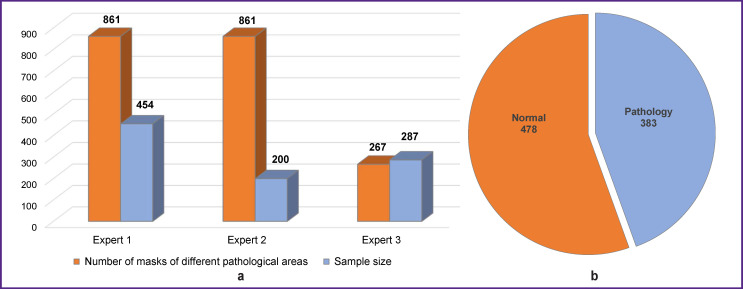
Sample structure for preliminary examination: (a) sample size and number of labelled masks; (b) sample partitioning (n=861) into “normal” and “pathology”

Two experts labelled 861 images each, one labelled only 267 images from the total sample (Figure 5). Each expert localized pathologies by labelling a mask at the image (more than one mask can be labelled at one image).

**Figure 6.**
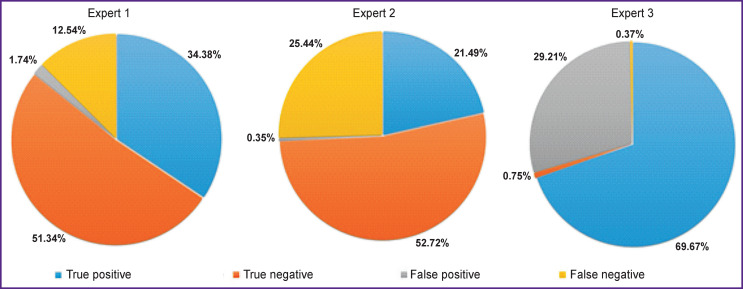
Results of arbitrage on binary conclusions

Binary arbitration results (presence of pathology/ no pathology) for each expert are shown at histograms (Figure 6). The regional level physician (expert No.1) showed the best results (73.27% sensitivity, 96.72% specificity); experts No.2 and 3 showed high specificity, 99.34 and 99.47%, respectively. These figures may be related to the physician’s specialization.

High sensitivity is required to diagnose image streams, i.e. with low pretest probability, e.g., in fluorographic screening. In mass screening, it is better to perform additional diagnostics on a healthy patient than to miss a patient with pathology.

High specificity is characteristic of image diagnosis tasks with high pretest probability. For example, patients with suspected pathology are admitted to the polyclinic X-ray room. Insufficiently justified treatment due to an erroneous conclusion in this case can lead to side effects and additional costs.

**Figure 7.**
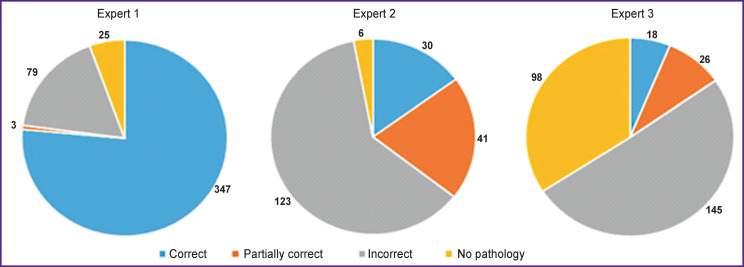
Arbitration results for labelling quality

Arbitration results by labelling quality are shown at histograms (Figure 7). All pathologies (masks) found by at least one physician were included in the analysis. “Correct” score for the mask was assigned when the arbitration concluded that the examiner performed the most accurate labelling. “Partially correct” score was assigned when the labelled pathology area matched the most accurate labelling by more than 50%. “Incorrect” score was assigned when the labelled pathology area overlapped with the most accurate labelling by less than 50% (or did not overlap at all). “No pathology” score was assigned when the examiner did not confirm the pathology at the image.

The analysis of the described study results demonstrated low level of convergence of physicians’ conclusions both on pathology presence or absence and on pathology localization at images. Therefore, in order to minimize the number of false labelling results, we proposed a method where three differently qualified physicians perform the reading and physicians of the highest qualification arbitrate their diagnoses.

Considering the results when opinion convergence of physicians with long experience was analyzed, we concluded that the primary data sample should be collected from diversified sources and a group of qualified specialists should label them. Low-quality images and those not meeting other technical criteria should be screened out.

A sample of about 300,000 medical images was used to train PhthisisBioMed service basic version. However, further research, including the above experiment and practical testing of the service, showed that the indiscriminate approach to training samples was inconsistent, and it was decided to switch to the quality-over-quantity approach. Thus, 9593 images from the initial sample sequentially selected automatically and manually were sent to qualified radiologists with at least 14 years of work experience for re-labelling. 4533 of these 9593 were evaluated as non-pathologic and 5060 as potentially pathologic. Some of the images were then removed from the sample as being controversial to the experts. The final baseline training sample scope was 8662 images, of which 2904 were normal and 5758 were pathological. Image labelling activities were conducted with participation of specialists from the Republican Clinical Anti-Tuberculosis Dispensary and other radiology specialists from polyclinics in Moscow and the Republic of Tatarstan (Russia).

### Validation

Validation (internal) should be a test on a sample selected in a special way: first, including all pathologies recognized by the MAI; second, including all normals. The default normal to pathology ratio should be close to 50/50 [5] or should correspond to a known ratio in the population which the AI services will be provided to [6].

The physicians who labelled the sample shall give a consolidated opinion on each image from the sample and develop the labelling jointly. All images with different opinions are discarded and replaced. After that, the reconciliation procedure is also carried out for the substituted images. The consilium of sample compilers must not coincide or overlap with the training sample compilers consilium. The author experience in the specialty must be at least 14 years.

A sample of 115 images was used for PhthisisBioMed service internal validation (at the time of writing): 52 without signs of pathology and 63 with various pathology signs (types of labelled pathology: 35 infiltrations, 4 pneumothoraxes, 23 pleural effusions, 4 foci, 2 disseminations, 13 calcinates, 3 cortical layer integrity violations).

To evaluate the service performance, standard metrics of diagnostic accuracy were used like area below characteristic ROC curve (AUCROC), sensitivity, specificity, etc.

The results significance is determined by the 95% confidence interval (CI). DeLong algorithm is used to calculate CI for AUCROC [7].

Acceptable sensitivity and specificity values are selected as standard by maximizing Youden index to maximize overall validity, or by maximizing the negative predictive value (NPV) to minimize false negatives.

**Figure 8.**
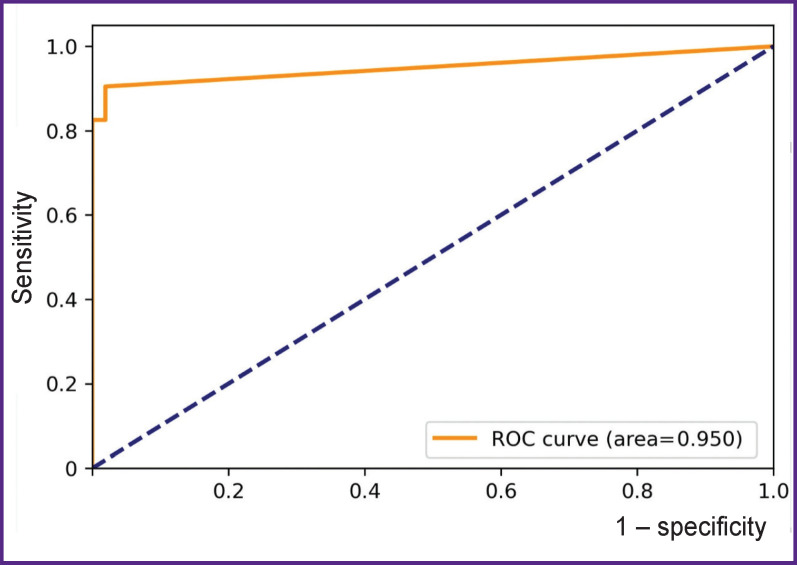
ROC curve built on the basis of 115 examinations set processed with the service (PhthisisBioMed service version 3.2)

The Youden index threshold is 0.79. The max NPV threshold is 0.79. Internal validation on the data set of 115 images achieved an AUCROC value of 95.0% (Figure 8).

### The Moscow experiment and external validation

At the beginning of 2020, the Moscow Department of Health announced it was starting to accept applications for participation in the “Experiment on the use of innovative computer vision technologies for medical image analysis and subsequent applicability in the healthcare system of Moscow” (hereafter — Experiment) [8]. The regulatory basis for the Experiment was Moscow Government resolution No.1543-PP dated November 21, 2019 and the related order of the Moscow Department of Health [9]. The aim of the Experiment was to scientifically investigate the possibility of using methods to support medical decision-making in the Moscow healthcare system based on the results of data analysis employing advanced innovative technologies. The Experiment was conducted on the basis of Moscow Unified Radiology Information Service (URIS UMIAS) platform uniting all medical organizations of the Moscow Department of Health (~2000 equipment units for radiology diagnostics of all modalities) [10]. According to the rules established by the organizers, legal entities could participate in the Experiment which provide services (software) based on computer vision technologies intended to analyze medical images for the following examination types:

chest computed tomography and low-dose computed tomography to detect lung cancer;

mammography to detect breast cancer;

lungs X-ray to determine lung pathology; chest computed tomography to detect new coronavirus infection.

On July 27, 2020, PhthisisBioMed service was admitted to URIS UMIAS and was put into streaming analysis of medical studies. In the early stages, activities were carried out together with the Experiment organizers to eliminate technical imperfections of information systems integration, to develop standardization, including mandatory completion of unified DICOM tags [11].

**Figure 9.**
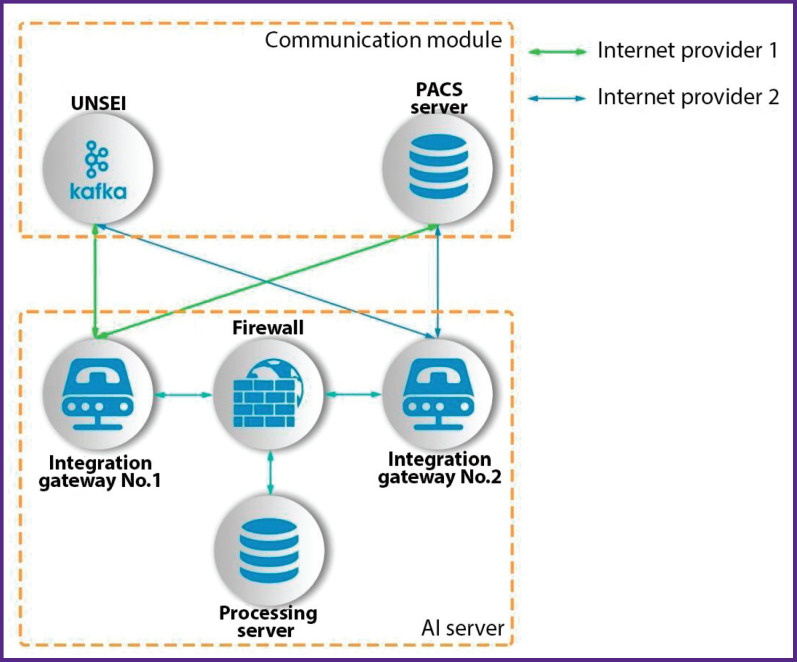
Hardware diagram of integration with URIS UMIAS

In order to solve technical problems and to improve the availability of the service for medical specialists, an interface was developed to integrate heterogeneous systems which included hardware and software gateways connected to URIS UMIAS via different Internet providers, which in turn solved the problems of hardware and software redundancy along with load sharing (Figure 9).

**Figure 10.**
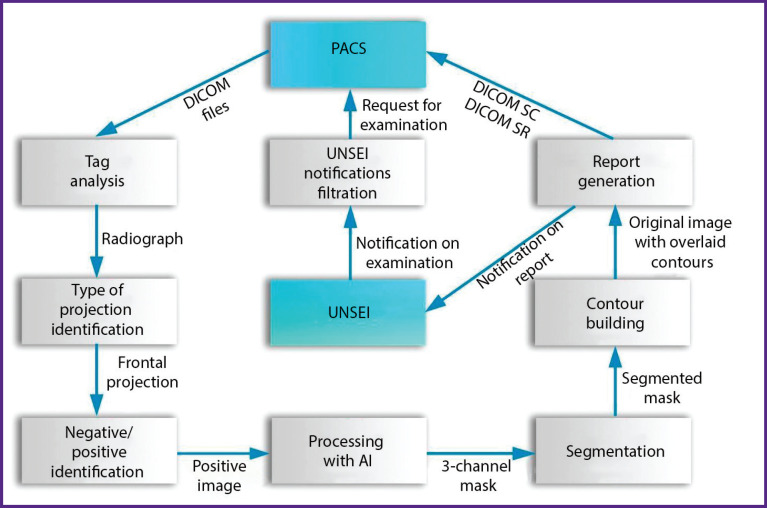
Functional diagram of integration with URIS UMIAS

URIS UMIAS architecture implies the interaction of AI services with two interfaces: unified notification system for external interactions (UNSEI) and the system of DICOM standard medical image transmission and archiving (PACS server). UNSEI is a URIS UMIAS subsystem designed to provide interaction and information exchange between URIS UMIAS nodes built on Apache Kafka platform [12]. This subsystem publishes in real time tasks for processing the examinations newly received in URIS UMIAS for all the subscribers connected to it. See Figure 10 for the functional diagram of the interface between the service and URIS UMIAS.

The load (stream of tasks to be processed) between the connected network nodes of one subscriber is automatically distributed by UNSEI. The system publishes tasks for processing studies for all subscribers connected to it. A filtering module was developed to ensure that the service could process only the actual studies intended for it (with recentness not exceeding 6.5 min to the moment of being processed by AI as the Experiment limitation). The software module reads and analyzes syntactically all UNSEI messages recording two examination identifiers: not outdated firstly, and intended for the service considering the patient modality (diagnostic equipment type) and age group secondly.

The hardware/software gateway requests and downloads study from the URIS UMIAS PACS server after parsing based on study ID.

The resulting DICOM study may contain a series of images with different projections (frontal and lateral), both negative and positive. To ensure that only correct images reach the AI processing, several successive checks are required:

analysis of DICOM tags characterizing the image modality;

image intelligent analysis using an auxiliary neural network to determine whether the submitted image is valid for analysis (i.e., whether the submitted image is a lung frontal radiographic projection in PhthisisBioMed service case);

classification of the image according to the negative/ positive criterion using a deterministic algorithm and negative image inversion.

The chest radiograph/fluorogram positive frontal projection is sent to the AI for processing and to the neural network which solves the task of lung contours segmentation. Then, if the AI concludes that a pathology is present, the contours of the detected pathology are plotted at the image. The contouring result is superimposed on the original image. Otherwise, the service gives the conclusion that no pathology is detected at the image. The last stage of medical image processing is classification of pathological signs. To solve this task, an auxiliary neural network (classifier) is used, and pathological areas identified during localization are fed to its input. These areas are extracted from the image and sequentially fed to the auxiliary neural network for analysis.

The classifier solves the classification task according to 9 pathological signs: pleural effusion, pneumothorax, atelectasis, dark focus, infiltration/consolidation, dissemination, cavity, calcinate/calcified shadow in lungs, violation of cortical layer integrity.

**Figure 11.**
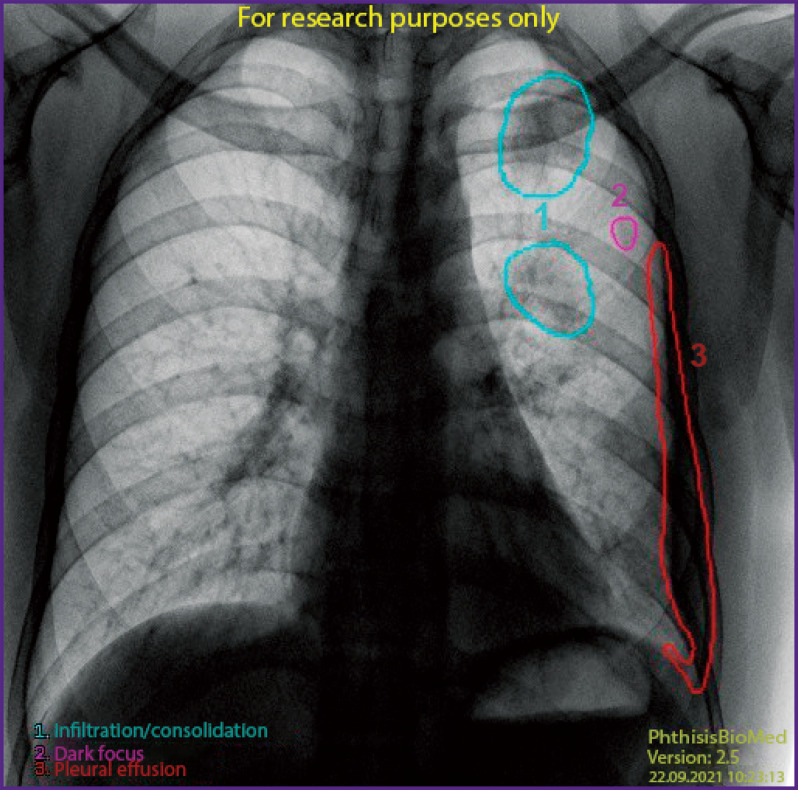
DICOM SC report

**Figure 12.**
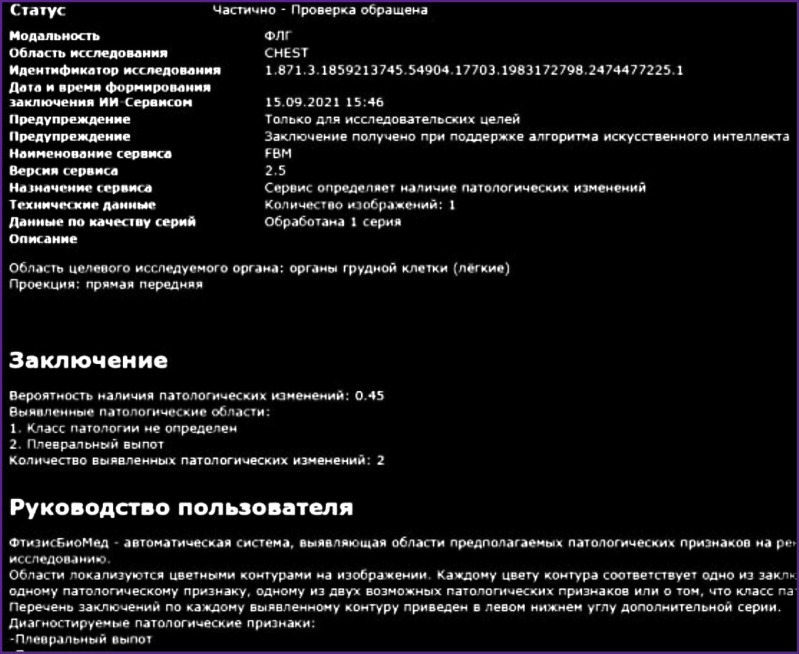
DICOM SR report

Based on the results of the examination processing with the service aid, a DICOM SC report (Figure 11) is sent to the radiological information system along with a DICOM SR report (Figure 12) and two messages to the UNSEI (Kafka) notifying that each DICOM report was sent.

At the final stage of integration into URIS UMIAS, a calibration test was conducted, and a decision was made to admit the service to the platform based on its results. See Table 1 for values of the service diagnostic consideration metrics [9] based on the results of the calibration test.

Several staging tests on the reference data set resulted in 0.965 AUCROC value, 0.92 sensitivity (95% CI: 0.87–0.97), 0.94 specificity (95% CI: 0.89–0.99). The Experiment procedures and the release of new versions of PhthisisBioMed AI service conditioned tests staging.

**Table 1:** Calibration test results

Metric	Obtained value on data sample	
	Youden index	max NPV
AUCROC	0.965	
Accuracy, 95% CI	0.93 (0.89–0.97)	0.93 (0.89–0.97)
Sensitivity, 95% CI	0.92 (0.87–0.97)	0.92 (0.87–0.97)
Specificity, 95% CI	0.94 (0.89–0.99)	0.94 (0.89–0.99)
Specific weight of false negative results (%)	0.08	0.08
Specific weight of false positive results (%)	0.06	0.06
Optimal threshold	0.65	0.65

Then, PhthisisBioMed AI service was admitted for use at examination streams. For automated analysis, the service was routed to the results of prophylactic chest radiological examinations performed at medical institutions of the Moscow Department of Health. In real clinical activities environment, PhthisisBioMed AI service showed its high quality and reliability confirmed with technological monitoring procedures (included to the Experiment methodology) and with the service inclusion into the top three of the monthly rating among the Experiment participants (https://mosmed.ai/ai/).

## Clinical trials of PhthisisBioMed AI service

### Research hypotheses

The tested hypothesis is formulated according to the format described by Korevaar et al. [13]. It is described as (with 95% CI) H_0_: {<0.93 sensitivity and/or <0.70 specificity}.

### Materials and Methods

The performed diagnostic study was retrospective. The design and results were described according to STARD 2015 methodology [14].

The test methodology [15] is based, but not limited to, on GOST R 59921.1—2022 “Artificial intelligence systems in clinical medicine. Part 1. Clinical evaluation”.

The index test was performed using the software “Program for automated analysis of digital chest radiographs/fluorograms according to TU 62.01.29001-96876180-2019” produced by PhthisisBioMed LLC, Russia (hereafter — PhthisisBioMed software). This software is intended for use by qualified employees of medical organizations. Its functionality allows to position it as a tool to support medical decision making. PhthisisBioMed software clinical implementation may potentially reduce the time of a doctor’s diagnosis, allowing to notice in time pathology signs and giving the doctor (medical organization employee) additional time to treat and rehabilitate the patient.

PhthisisBioMed software is indicated to analyze digital fluorographic images (X-rays) of lungs in anterior-posterior projection and to detect pathologies.

Radiological signs of the following pathologies are detected automatically:

1. Type 1 pathologies — conditionally “dangerous”. Signs: >1.5 cm infiltration (focus); cavity; pneumothorax; hydrothorax; focus; pathological changes of lung roots; fluid level; foci.
2. Type 2 pathologies — conditionally “not dangerous”. Signs: interstitial changes in pulmonary parenchyma; cirrhosis; fibrothorax; changes in pleura; calcinates/ calcification; diaphragmatic hernia; changes in bones; chains of metal sutures; foreign bodies; area of increased transparency (not cavity); atelectasis; changes in mediastinal organs.

No absolute or relative contraindications to the software use exist.

The reference test is a verified reference labeled data set [16].

To form the initial data set, we used fluorographic examination results from URIS UMIAS in accordance with the current legislation. All examination results were depersonalized in accordance with the established procedure.

Initial data were selected from URIS UMIAS according to the following parameters: procedure description, diagnostic device type, medical organization type, image date, patient age. About 400,000 examination identifiers were obtained as selection result.

Radiologists’ reports were obtained from URIS UMIAS for the selected examinations. These reports were subsequently analyzed by key words. Only fluorograms of patients over 18 years old were included to the analysis. As a result of key word selection, a data set of 5000 images was obtained for further verification and use in the study of diagnostic accuracy of PhthisisBioMed software.

The data set includes the results of fluorography: diagnostic images without pathology signs (normal) and images with all radiological pathology signs listed above.

After the selected fluorographic findings were analyzed visually, the following was excluded: questionable images without evident pathology signs listed at the manufacturer’s documentation and which could not be reliably classified as “normal”; images of insufficient technical quality (low contrast, etc.).

The selection resulted in 1536 images containing only frontal images of the chest area. Since the tested software cannot process lateral projections according to its documentation, these images were forcibly removed during the verified data set compilation.

The final data set included results of examinations performed on diagnostic machines of 18 different models (11 manufacturers), 4 of which fell into fluorographer category (n=670), the remaining 14 fell into X-ray diagnostic machine category (n=866). Next, the data set was labelled.

Four radiologists with 9 to 40 years of experience participated in data set labelling and verification. Two radiologists who analyzed frontal projections labelled the data set. One or more radiological signs of pathology were compared with each image during inspection. Images with no signs of pathology were categorized as “normal”.

According to the analysis results, 393 images out of 1536 contained no pathology signs. Other 1143 images contain labels of one or more pathological signs (in total, 3304 sign labels on the whole group of images with pathology signs). Of these, 291 images contain only one label each, and thus these images can be accurately assigned to a particular class of pathology signs.

The data set was verified by two radiologists with advanced degrees and certificates of good clinical practice.

### Statistical analysis

The required sample size and statistical significance were calculated according to standard methods [17].

Diagnostic accuracy metrics (sensitivity and specificity) were calculated using the methodology recommended for clinical trials based on AI software [17].

The software accuracy was determined in two stages: the first stage assessed the system integral characteristic without division into radiological signs, and the second stage assessed individual radiological signs accuracy.

The statistical significance of the results was determined by 95% CI.

### Results

The verified data set (n=1536) was processed using PhthisisBioMed software. Average processing time per image did not exceed 5 s. Textual reports were obtained for 1517 images (98.8%) containing information on pathology presence probability (p∈[0, 1]) in each particular image.

No textual reports were obtained for 19 images. Therefore, we attempted to process them separately. Textual reports were neither obtained during the second processing, so these images were excluded from the verified data set list and were not analyzed further.

Thus, the refined verified data set contains 1517 images. Of these, 391 (25.8%) contained no signs of pathology. See Table 2 for distribution of radiological signs.

**Table 2:** Test data set of images with signs of pathology (n=1126) successfully processed using PhthisisBioMed software

Radiological sign	Number of images containing only this sign	Number of images containing this sign along with other signs
Total (subtotal) darkening (compaction) of the lung field, including:	2	80
infiltration	1	48
fluid level	1	32
Limited (lobular, segmental, subsegmental, multisegmental) lung dark field (infiltration), including:	18	348
infiltration	15	186
fluid level	1	126
fibrothorax	1	20
cirrhosis	1	16
Circular shadow of lung localization	33	87
Ring-shaped shadow, including:	20	110
diaphragmatic hernia	14	44
cavity	6	66
Air in pleural cavity	2	74
Foci	17	214
Focal disseminations	1	79
Strengthening and deformation of the pulmonary pattern (interstitial changes in the pulmonary parenchyma, pneumofibrosis, pneumosclerosis)	76	755
Pleural thickening	18	300
Dilation (± deformation) of lung root shadow/roots or displacement of lung root shadow/roots	4	283
Calcinates/calcification of pulmonary and extrapulmonary localization	17	147
Traumatic changes of bone structures	24	79
Foreign bodies	34	216
Change of mediastinal shadow	22	438
Site of increased transparency	1	49
Labels total	289	3259

Accordingly, 1126 images (74.2%) contained 3259 pathology labels. Only 289 images contained one pathology label; 837 images contained more than one label per image (2970 labels, an average of 3 or 4 labels per image).

**Figure 13.**
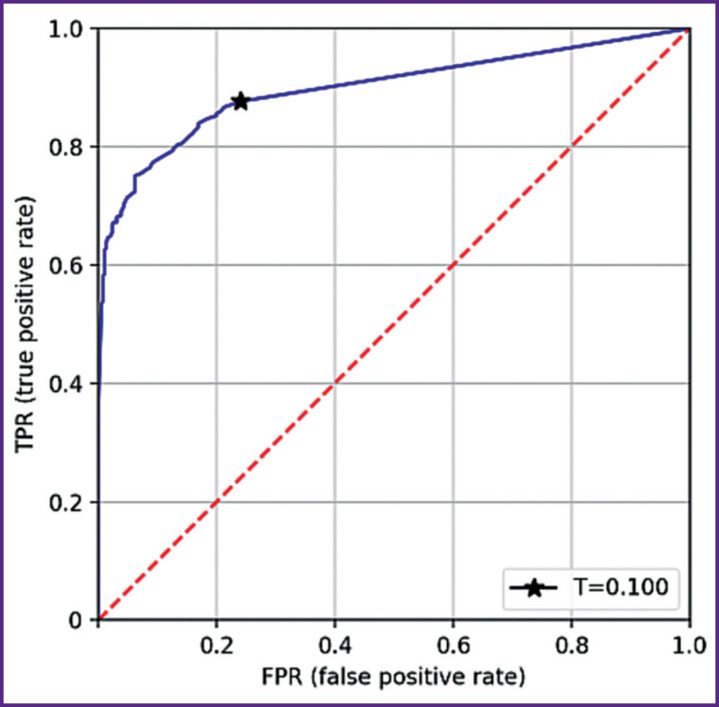
ROC curve of data set analysis without splitting into signs (0.1 activation threshold condition)

The number of true positive, true negative, false positive, false negative results was determined, a four-field table was created and analyzed along with a characteristic curve (ROC curve, Figure 13), and T*=*0.1 optimal value was established of activation threshold (cut-off), the value against which maximum sensitivity was achieved with specificity not less than 0.3 was considered optimal. At the first stage, integral estimation was applied to accuracy indicators, and at the second stage — to individual radiological signs.

#### Evaluation of sensitivity and specificity integral indices

For a given threshold, we obtained the following values of accuracy indicators: 0.876 (95% CI: 0.85–0.89) sensitivity; 0.75 (95% CI: 0.71– 0.78) specificity.

All 94 false positive and 139 false negative cases were referred to radiologists for re-examination. The re-labelling was “blind”: no additional information on previous labelling of these images and no information on results of processing with PhthisisBioMed software was given to the physicians.

Disputed cases were evaluated expertly by working group members with a degree in medicine and relevant specialization. The experts confirmed that all cases not recognized by the software were indeed correctly classified as “normal” or “pathology” during the initial labelling. Thus, all recognition errors were attributed to incorrect operation of PhthisisBioMed software.

#### Evaluation of indexes based on individual radiological signs

Since the number of unique images was insufficient to confirm most signs, an iterative approach was used:

1. Radiological signs were selected with sensitivity not reaching the threshold value according to test results.
2. Among them, the sign was selected contained in the largest number of unique images (this was “Pulmonary pattern enhancement and deformation” sign (n=76) in the first iteration).
3. Sensitivity was determined for the selected group of images. The index was 0.81. On this basis, it was recognized that the diagnostic sensitivity of the above sign is insufficient.
4. 76 images were excluded from the analysis being unique by this sign.
5. This sign was excluded from the analysis. Besides, the images that previously included this sign along with some other sign, were now considered to include only the other sign.
6. After excluding the images and the sign selected in step 2, we returned to step 1.
7. Steps 1–6 were repeated until the integral sensitivity of the remaining signs reached the 0.93 value declared by the manufacturer. 7 signs were excluded (annular shadow (diaphragmatic hernia); lung pattern enhancement and deformation (interstitial changes in lung parenchyma, pneumofibrosis, pneumosclerosis); dilation (deformation) of lung root/roots shadow or shift of lung root/roots shadow; foreign bodies; mediastinal shadow changes; circular lung localization shadow; area of increased transparency) and 371 false negative tests.

Table 3 presents the signs with 0.93 integral sensitivity.

**Table 3:** Signs with ≥0.93 integral sensitivity

Radiological sign	Number of images	Sensitivity P1 by sign	95% CI P1	significance Statistical α at P_0_=0.93
Total (subtotal) lung dark field (compaction), including:				
infiltration	48	1.0	0.997–1.0	0
fluid level	32	1.0	0.996–1.0	0
Limited lung dark field, including:				
infiltration	186	0.989	0.97–1.0	10^–14^
fibrothorax	20	1	0.996–1.0	0
cirrhosis	16	1	0.995–1.0	0
fluid level	13	0.923	0.78–1.0	-
Air in pleural cavity	74	0.986	0.959–1.0	4.4×10^–5^
Foci	214	0.958	0.93–0.98	0.04
Focal disseminations	79	1	0.998–1.0	0
Pleural thickening	300	0.97	0.95–0.98	4.9×10^–5^
Calcinates/calcification of pulmonary and extrapulmonary localization	147	0.986	0.97–1.0	7.7×10^–9^
Traumatic changes of bone structures	41	0.90	0.80–0.99	—
Ring-shaped shadow (cavity)	13	0.923	0.78–1.0	—

1. was rejected for the following signs: infiltration — at partial and total darkening; hydrothorax (fluid level) — at partial and total darkening; cirrhosis; fibrothorax; atelectasis — at partial and total darkening; foci; cavity; pneumothorax; foci (dissemination); pleural changes; calcinates; changes in bone structures;
2. was accepted for the following signs: infiltration (focus); diaphragmatic hernia; interstitial changes in lung parenchyma; pathological changes in lung roots; metal suture chains, foreign bodies; changes in mediastinal organs; area of increased transparency (not cavity).

## Particular examples of analysis

To clarify aspects of the study, let us formulate a few theses.

1. AI is not guided by auxiliary factors other than those that are presented to it, and even of these, it operates only with those which it is capable to interpret. In other words, if the AI is designed to analyze images, that is all it will do. By default, age, sex, blood test results, patient history, and other factors are not taken into account. If more advanced models or algorithmic superstructures are used, some of these factors may be considered. However, universality and wide coverage of analyzed attributes often come at the expense of system accuracy. In the same way, when AI is adapted to solve more than one task, e.g. localization and classification tasks, the same price would be paid.
2. The AI opinion is a mathematical result, so it is often highly subjected to distortion depending on the conditions of analysis performing. The initial data are very important. Distorted, noisy images or partial images of the analyzed organs lead to inference “garbage” results. For example, proper patient positioning is of paramount importance for AI analysis of radiographic images. In addition, even by analyzing images of the same patient taken with different X-ray machines or even with the same X-ray machine but with different settings, MAI may give slightly different conclusions.
3. No matter how many times PhthisisBioMed AI service analyzes the same examination at the same conditions, it will come to the same conclusion within the same training iteration.

In view of the above, we suggest paying attention to several results of processing examinations of real patients using AI.

### Example 1

**Figure 14.**
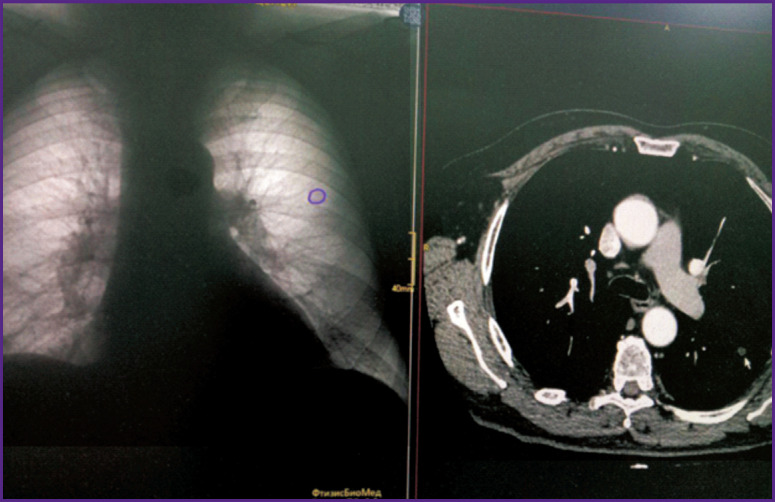
Prophylactic fluorography processing using PhthisisBioMed service (*left*) and CT scan results (*right*)

*PhthisisBioMed AI revealed an 8-mm diameter rounded neoplasm or tuberculous focus at a prophylactic fluoroscopy (Figure 14). Further follow-up examination in the form of CT scan with intravenous contrast showed the absence of tuberculosis and excluded the malignancy of the mass. The most probable diagnosis based on CT scan was “benign congenital mass — lung hamartoma” (with no pathological tumor vascular network feeding the mass).*

### Example 2

**Figure 15.**
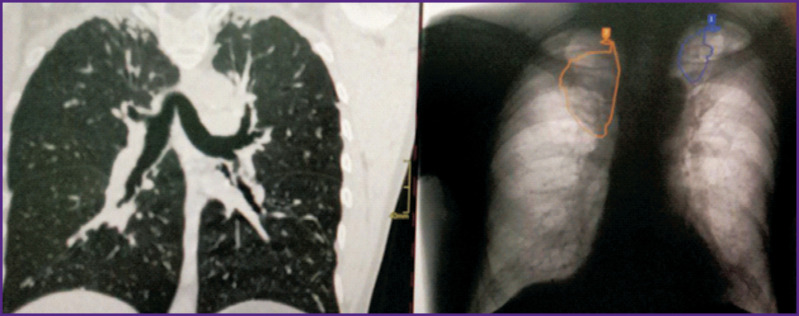
Fluorography stream processing using PhthisisBioMed service (*right*) and CT scan results (*left*)

*PhthisisBioMed AI found changes at the fluorography (Figure 15) on both lungs apices. To rule out active tuberculosis, the physician referred to the fluorographic archive: no dynamics was found. An earlier CT scan revealed massive pleuroapical layering on both sides. No active tuberculosis was diagnosed.*

### Example 3

**Figure 16.**
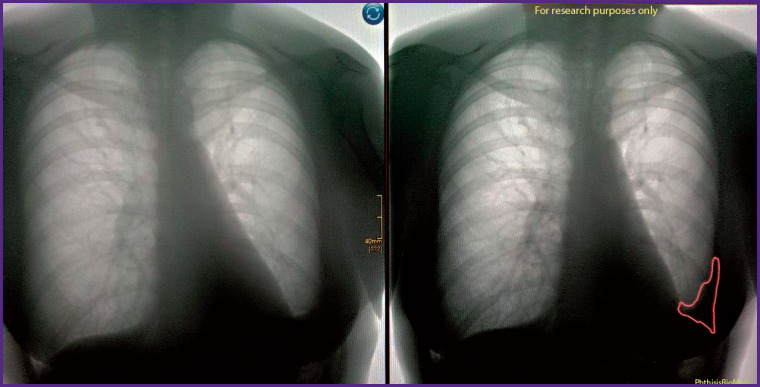
Fluorography stream processing using PhthisisBioMed service (*right*) and original image (*left*)

*PhthisisBioMed AI showed darkening of the lateral pleural sinus at the left side (Figure 16). First of all, it is necessary to exclude inflammatory fluid presence in the pleural cavity. This female patient had surgery with partial lung resection, which caused elevation of the diaphragm on the left, shortening and darkening of the sinus on the left. The medial structures (heart and vessels) were markedly displaced to the left as the lung volume was reduced after surgery. Taking into account the history of surgery and stable radiological picture for three months, it can be concluded that inflammatory fluid in the pleural sinus at the left side was excluded.*

### Example 4

**Figure 17.**
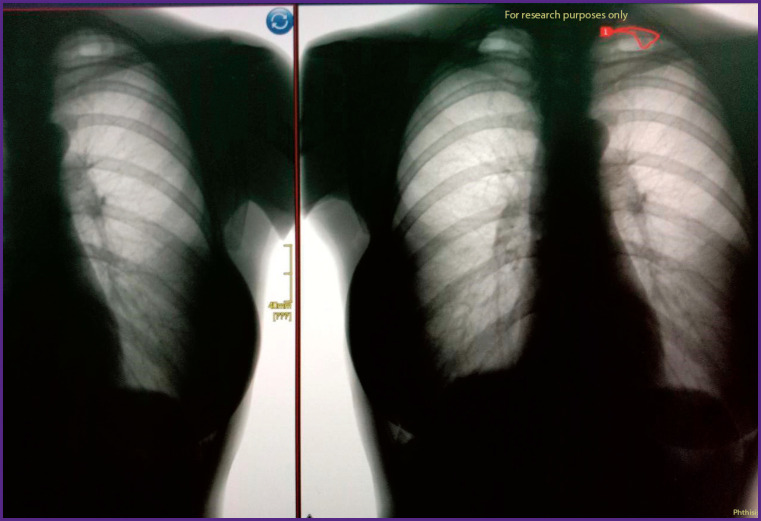
Detection of congenital cervical rib with the help of PhthisisBioMed artificial intelligence (*right*) and original image (*left*)

*PhthisisBioMed AI labeled at fluorography (Figure 17) a change of a congenital cervical rib form at the left side (it is more often a compensated pathology, but occasionally neurological symptoms may be present).*

### Example 5

**Figure 18.**
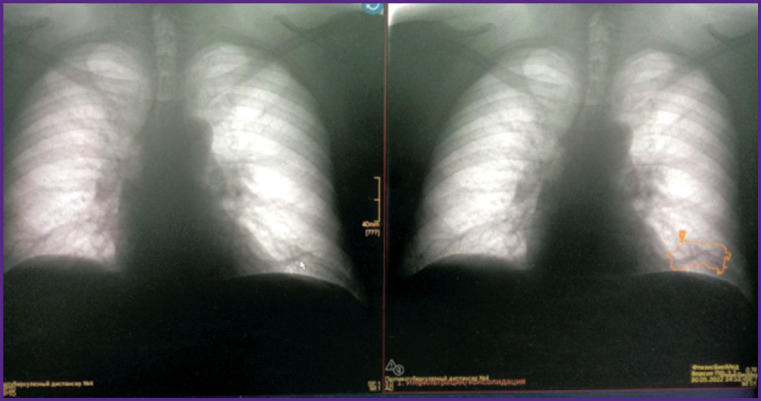
Detection of pneumonia consequences with the help of PhthisisBioMed artificial intelligence (*right*) and original image (*left*)

*PhthisisBioMed AI revealed at fluorography (Figure 18) consequences of pneumonia in the form of linear local deformation of the lung pattern in the basal part of the left lung. According to the database, these cicatricial changes remained without dynamics during 6 months.*

### Example 6

**Figure 19.**
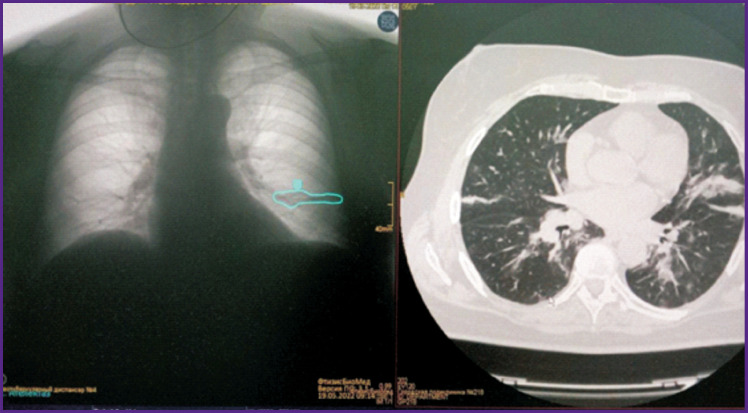
Detection of discoid atelectasis with the help of PhthisisBioMed artificial intelligence (*left*) and CT scan results (*right*)

*PhthisisBioMed AI labeled at fluorography (Figure 19) an area of linear deformation of the lung pattern and suggested differentiation between disc-shaped atelectasis and postpneumonic fibrosis. A CT scan from the archive shows the extent of lung lesion CT3 at COVID-19 two years prior to the present. Conclusion: postpneumonic fibrosis at fluorography (although even incomplete dissection of lung area with formation of fibroatelectasis after massive inflammation with formation of rough scar changes, fibrosis in lungs is possible).*

### Example 7

**Figure 20.**
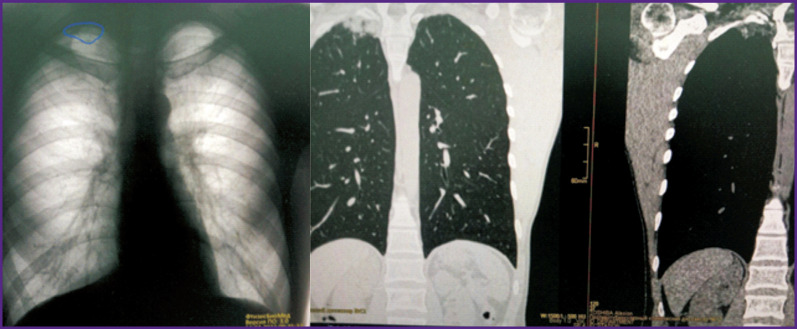
Detection of residual changes with the help of PhthisisBioMed artificial intelligence (*left*) and CT scan results (*right*)

*PhthisisBioMed AI revealed significant residual changes after a tuberculosis history (Figure 20). Further CT examination confirmed the changes and ruled out infection activity.*

The results of the present study showed that because physicians’ diagnostic metrics can be highly heterogeneous due to their specialization and experience, using an individual physician’s opinion as a benchmark opinion when evaluating AI diagnostic metrics is not appropriate. An objective evaluation requires collective opinion.

The nature of physician and AI errors is different, while the metrics are comparable. On the one hand, AI errors are often obvious to the physician; on the other hand, the AI service can focus the physician’s attention on a non-obvious pathological area that could potentially have been missed by the physician. Combining the physician and MAI into a system can generate a synergistic effect, expressed as an increase in the diagnostic metrics of the physician + MAI system relative to the metrics of the physician and MAI separately.

Studies conducted both within the framework of the Moscow experiment (more than 8 million medical images processed by 2023) and by international experts in recent years clearly demonstrate that the quality of AI analysis of medical images is close to the quality of experienced diagnosticians work, and this allows to look into the future of AI technologies with cautious optimism. As a result of a long period of research, development, and testing, the viability of CDSS based on AI technologies was revealed and proved in practice.

To develop and increase the effectiveness of MAI technologies, methodologies for training and testing AI products were developed and tested in practice, and MAI was implemented into the clinical processes of medical institutions.

## Conclusion

As part of the Moscow experiment, PhthisisBioMed AI service underwent stepwise testing procedures on reference data sets. High values were achieved: 0.965 area below characteristic ROC curve, 0.92 (95% CI: 0.87– 0.97) sensitivity, 0.94 (95% CI: 0.89–0.99) specificity. In real clinical activities environment (result streams processing of preventive chest radiological examinations at medical organizations of the Moscow Department of Health), PhthisisBioMed AI showed its high quality and reliability confirmed with technological monitoring procedures (included to the experiment methodology).

Clinical trials of PhthisisBioMed AI service were conducted on a verified data set (n=1536) considering epidemiological indicators of the thoracic organs major diseases prevalence. In the process of testing, it was found that PhthisisBioMed AI diagnostic accuracy was unequal for different pathological signs; therefore, several of these signs were excluded from the AI service capabilities. As a result of clinical tests, the average sensitivity of PhthisisBioMed medical AI service was determined as 0.975 (95% CI: 0.966–0.984).

PhthisisBioMed is registered as a medical device (Registration Certificate of the medical device No.RZN 2022/17406 of May 31, 2022) and is actively used in the Russian Federation as a diagnostic tool to reduce the burden on the radiologist and expedite the process of obtaining a medical report.
